# Estimation of hospital-based HIV seroprevalence as a nationwide scale by novel method; 2002-2008 in Korea

**DOI:** 10.1186/1471-2458-10-739

**Published:** 2010-11-30

**Authors:** Jin-Hee Lee, Kee-Jong Hong, Jin-Sook Wang, Sung Soon Kim, Mee-Kyung Kee

**Affiliations:** 1 Division of AIDS, Korea Centers for Disease Control and Prevention, Seoul, Korea; 2 Division of Influenza Viruses, Korea Centers for Disease Control and Prevention, Seoul, Korea

## Abstract

**Background:**

In Korea, approximately 70% of HIV-positive individuals are currently diagnosed in hospitals, while most HIV-positive patients were diagnosed at public health centers in 1980 s and 1990 s. However, there are no reporting systems to identify how many HIV tests are performed in the Korean hospitals different from public health centers and Blood centers. We estimated how many HIV tests were performed in hospitals and analyzed the nationwide hospital-based HIV seroprevalence in the present study.

**Methods:**

Between 2002 and 2008, data included HIV tests on insurance claims in hospitals and the proportion of computerized insurance claims from the Health Insurance Review and Assessment Services. The number of HIV tests from the survey in the External Quality Assurance Scheme for hospital laboratories was collected to calculate the insurance claim proportion. HIV seroprevalence was estimated using data of tested individuals, including infected individuals. Statistical analysis was confirmed with the 95% confidence interval. Statistical significance was defined at p-values < 0.05.

**Results:**

The number of HIV tests in hospitals increased from 2.7 million in 2002 to 5.0 million in 2008. The trend of HIV seroprevalence was decrease (1.5-1.3 per 10,000 individuals, P < 0.0028), except in 2002. The number of women tested was greater than men, and the proportion increased in older individuals and in small towns. Men had a higher annual HIV seroprevalence than women (P < 0.0001). The annual seroprevalence decreased in men (P = 0.0037), but was stable in women. The seroprevalence in the 30-39 year age group demonstrated higher than other age groups except 2008.

**Conclusions:**

The nationwide hospital-based number of HIV tests and seroprevalence were estimated using a new method and seroprevalence trends were identified. This information will facilitate improvement in national HIV prevention strategies.

## Background

UNAIDS has provided basic information about the HIV/AIDS epidemic by predicting the number of HIV-infected individuals in each country, in addition to the worldwide data [1,2]. The AIDS Epidemic software (EPIMODEL) was developed by the WHO in the late 1980 s to estimate the past and current HIV prevalence, and to make short-term projections of AIDS cases and deaths in areas where AIDS case reporting was largely incomplete and unreliable [3,4]. UNAIDS reported that there are 33.4 million people living with HIV/AIDS (PLWHA) worldwide, and 4.7 million HIV-positive individuals were alive in Asia in 2008 [5]. There is an enormous difference in the national HIV prevalence among Asian countries [6]. Specifically, south and southeast Asia have a high prevalence, while Korea and Japan have a low prevalence [7].

A total of 6888 cumulative HIV infections were identified in Korea as of 2009; 82% (5671) of these individuals had been previously living with HIV/AIDS. The number of newly diagnosed individuals is increasing annually [8]. It is unclear whether the increase is secondary to increased transmission rates or to increased testing and identification. We hoped to estimate the annual number of HIV tests nationwide and compare this to the annual HIV prevalence. However, the nationwide seroprevalence and total number of HIV tests has not been estimated because the number of HIV tests in the hospitals was lacking.

The number of HIV tests in public health centers (PHCs) decreased due to changes in strategies for HIV testing from 1998 in Korea [9]. In contrast, the number of HIV tests in hospitals has increased since 2000 due to increased medical examinations, including regular health check-ups, tests for pregnant women, and pre-surgical testing. The current proportion of new HIV diagnoses in hospitals is 70% [10]. PHCs and blood centers manage data of HIV testing through a computerized system and regularly report the number of HIV tests to the Korea Centers for Disease Control and Prevention (KCDC) [11-13]. However, there are no similar reporting systems to identify how many HIV tests are performed in the greater than 5000 Korean hospitals [14]. Hospital-derived testing data is very important in the estimation of nationwide HIV seroprevalence, and prevalence models or estimation methods are required to estimate the number of HIV tests performed in the hospital.

The nationwide HIV seroprevalence is important for AIDS prevention and evaluation of HIV testing strategies. We therefore estimated the number of HIV tests and HIV seroprevalence in hospitals. We developed a method to estimate the number of HIV tests performed in hospitals, calculated HIV seroprevalence in the hospital setting, and analyzed the trends in HIV seroprevalence.

## Methods

### Data collection

#### Health insurance claims for HIV testing

**Figure 1:**
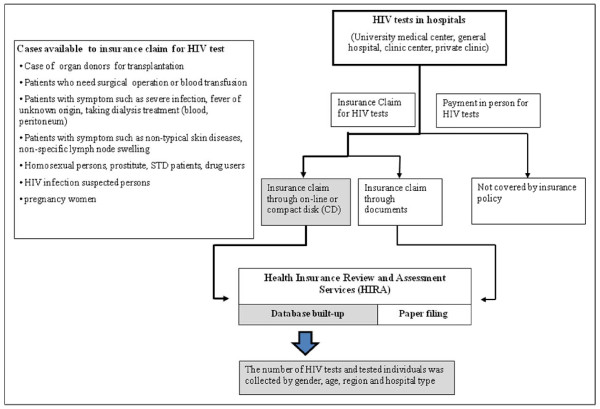
**Flowchart of insurance claim for HIV testing in hospitals**.

National medical insurance is required for every citizen of Korea. Hospitals claim medical costs to the Health Insurance Review and Assessment Services (HIRA). Medical costs are usually subsidized by the government when HIV tests are performed for specific indications (summarized in Figure 1 revised on 26 December 2008) [15], and insurance claim data for HIV tests are collected from HIRA to estimate the number of HIV tests performed in hospitals. Claims for medical costs to HIRA are processed on-line, or via compact disks (CDs) or documents. Among the data, claims processed via documents are not computerized. Computerized proportions by year and hospital type are collected to adjust non-computerized claims (Table 1). The number of yearly HIV tests and the number of individuals who claimed insurance for HIV tests are collected by gender, age, region, and hospital type during 2002-2008. Regions are classified as metropolitan or small town [11]. Hospitals are classified into four hospital types, as follows: university medical center, general hospital, clinic center, and private clinic.

**Table 1:** Computerization of insurance claim for HIV testing and the number of HIV tests per individual by year and hospital type (2002-2008)

Hospital type	Item	2002	2003	2004	2005	2006	2007	2008	P-value*
University medical center	Computerization (%)^1)^	89.6%	96.5%	99.1%	100%	100%	100%	100%	
	Repeated value^2)^	1.046	1.048	1.048	1.045	1.055	1.059	1.069	0.0120

General hospital	Computerization (%)	53.4%	73.4%	84.2%	94.8%	98.4%	97.7%	98.1%	
	Repeated value	1.028	1.030	1.032	1.029	1.037	1.057	1.050	0.0173

Clinic center	Computerization (%)	25.9%	52.7%	71.5%	91.3%	96.7%	96.2%	98.4%	
	Repeated value	1.021	1.024	1.030	1.030	1.041	1.044	1.042	0.0008

Private clinic	Computerization (%)	87.9%	90.7%	92.7%	94.8%	95.2%	95.3%	95.4%	
	Repeated value	1.091	1.103	1.112	1.104	1.101	1.102	1.077	0.4077

^1)^Data from Health Insurance Review and Assessment Services.

^2)^The number of HIV tests per individual for a year.

University medical center: Hospital with greater than 100 beds that provide more than 8 services of medical examination of patients with an associated medical school.

General hospital: Hospital with greater than 100 beds that provide more than 8 services of medical examination of patients without an associated medical school.

Clinic center: Hospital with 30-100 beds.

Private clinic: Private clinic with less than 30 beds that provide medical examination primarily on an outpatient basis.

*P-value for trend test of repeated value.

#### The number of HIV tests by hospitals

For evaluation of proficiency in HIV tests, the External Quality Assurance Scheme (EQAS) has been performed for HIV testing laboratories every 6 months by the Division of AIDS, with KCDC as the HIV national reference laboratory. The number of HIV tests in hospital laboratories was surveyed through EQAS in 2005, 2006, and 2008 [16,17]. Therefore, we collected the number of yearly HIV tests in each hospital in 2005, 2006, and 2008 from HIRA to estimate the insurance claim proportions. Also, the number of HIV tests in private clinics was collected from 24 main commercial clinical laboratories because private clinics usually referred HIV testing to the commercial clinical laboratories.

#### HIV-infected individuals

HIV-reactive cases from hospital as screening sites were referred to the Division of AIDS at KCDC or 17 local sites for the Institute of Health and Environment for confirmation of HIV infection. Cases confirmed as positive were reported to the Division of HIV and TB control at KCDC, which manages the HIV Database. Hospital laboratories usually use antigen/antibody Enzyme Linked Immunosorbent Assay (ELISA) test, Particle Agglutination Test (PA), or Rapid test for HIV screening. Confirmation institutes use antigen ELISA, antibody ELISA, PA, and Western Blot for confirmation of HIV infection [18]. We collected HIV cases data from the Division of HIV and TB control during 2002-2008 for the estimation of HIV seroprevalence in the present study.

### Statistical methods

#### Assumptions for estimation of HIV seroprevalence

HIV seroprevalence was defined as the number of HIV-infected individuals divided by the total number of HIV-tested individuals in a single year during the study period. HIV seroprevalence was estimated under several assumptions. First, the insurance claim proportions for HIV testing by hospital type are similarly distribute in replying and non-replying hospitals. Second, the insurance claim proportions for HIV testing in the years without a survey (2002-2004, and 2007) are similar to the nearest year with a survey (2005, 2006, and 2008). Third, as the proportion between the number of cases claimed for HIV testing and the number of individuals claiming insurance, the repeated value for an individual with an insurance claim is similar to the repeated value for individuals without an insurance claim.

#### Insurance claim proportion

In the years with a survey (2005, 2006, and 2008), only hospitals with both the number of insurance claims for HIV tests from HIRA and the number of HIV tests by the HIV EQAS survey were classified by hospital type. Insurance claim proportions for HIV testing were calculated as the number of insurance claims divided by the number of HIV tests. However, the insurance claim proportions for years without a survey (2002-2004, and 2007) were estimated. The insurance claim proportions for HIV testing during 2002-2004 were estimated from 2005. The proportion in 2007 was estimated as the mean of 2006 and 2008.

#### The number of HIV tests and the number of HIV-tested individuals

The number of HIV tests was estimated as the number of insurance claims with division by insurance claim proportion and division by the computerized proportion of insurance claims for HIV tests. The number of HIV-tested individuals was calculated as the number of HIV tests divided by the repeated value which was calculated in each year by using the number of HIV tests divided by the number of HIV-tested individuals from HIRA.

#### Estimation of HIV seroprevalence

Finally, HIV seroprevalence was represented as the number of HIV-infected individuals per 10,000 HIV-tested individuals in 1 year. The estimation of seroprevalence by gender, age, and region was also performed.

#### Statistical analysis

The mean of repeated values by hospital types was compared using ANOVA. The linearity of the repeated value and the number of insurance claims by year in hospital types was analyzed by trend tests. SAS 9.1 software was used for the entire evaluation procedure. Statistical analysis was confirmed with the 95% confidence interval (CI). Statistical significance was defined at p-values < 0.05.

## Results

The proportion of computerized insurance claims was currently more than 95% (Table 1). General hospital and clinic centers have increased for computerization of insurance claims from 53.4% and 25.9% in 2002 to 98.1% and 98.4% in 2008, respectively. The repeated values were the highest in private clinics (P < 0.0001); however, the annual repeated value of private clinics was not increasing (P = 0.4077).

The estimated number of HIV tests and insurance claim proportions in hospitals are summarized, as shown in Table 2. The total number of HIV tests in hospitals increased from 2.8 million in 2002 to 5.0 million in 2008, and also the number of insurance claims for HIV tests increased annually in all hospital types (P < 0.0001). The highest insurance claim proportion for HIV tests was from university medical centers, followed by general hospital and clinic centers.

**Table 2:** Estimation of insurance claim proportion for HIV testing and the number of HIV test based on hospital type in Korea (2002-2008)

Hospital type	Item	2002	2003	2004	2005	2006	2007	2008
University medical center	No. of institutes^†^	42	42	43	42	43	43	43
	No. of institutes participating in survey (%^‡^)*	-	-	-	38 (90.5%)	40 (93.0%)	-	41 (95.3%)
	Insurance claim proportion for HIV test^§^	33.9%	33.9%	33.9%	33.9%	39.7%	43.1%	46.5%
	No. of insurance claims	188 006	232 114	268 914	311 852	408 904	480 238	560 928
	Estimated number of HIV tests	618 962	709 536	800 461	919 917	1 029 985	1 114 241	1 206 297

General hospital	No. of institutes	241	241	163	215	242	249	268
	No. of institutes participating in survey (%^‡^)	-	-	-	143 (66.5%)	164 (67.8%)	-	141 (52.6%)
	Insurance claim proportion for HIV test	21.1%	21.1%	21.1%	21.1%	24.9%	31.7%	38.5%
	No. of insurance claims	96 636	154 322	202 994	292 957	380 878	492 621	572 374
	Estimated number of HIV tests	857 660	996 436	1 142 585	1 465 580	1 554 503	1 590 593	1 515 480

Clinic center	No. of institutes	783	871	314	470	573	759	888
	No. of institutes participating in survey (%^‡^)	-	-	-	63 (13.4%)	77 (13.4%)	-	78 (8.9%)
	Insurance claim proportion for HIV test	16.8%	16.8%	16.8%	16.8%	15.9%	19.2%	22.4%
	No. of insurance claims	40 261	80 111	107848	134 546	189 783	250 976	330 385
	Estimated number of HIV tests	925 285	904 841	897 835	877 184	1 234 337	1 358 801	1 498 916

Private clinic	No. of institutes	22 760	23 559	1913	2028	2267	2396	2448
	No. of institutes participating in survey (%)**	-	-	-	22 (1.1%)	24 (1.1%)	-	23 (0.9%)
	Insurance claim proportion for HIV test	11.0%	11.0%	11.0%	11.0%	11.4%	12.4%	13.3%
	No. of insurance claims	34 862	45 544	46 475	57 637	74 952	91 242	104 338
	Estimated number of HIV tests	360 555	456 490	455 771	552 714	690 624	772 112	822 323

Total	No. of insurance claims	359 765	512 098	626 231	796 794	1 054 517	1 315 077	1 568 025
	Estimated number of HIV test	2 762 462	3 067 303	3 296 653	3 814 395	4 509 448	4 835 747	5 043 015

^†^No. of institutes: Number of institutes claiming medical care fee for HIV testing to Health Insurance Review and Assessment Services (HIRA).

^§^Insurance claim proportion for HIV tests in years with survey (2005-2006, 2008). Without survey, insurance claim proportions for HIV tests during 2002-2004 were estimated by that of 2005 and the proportion in 2007 was estimated as the mean of 2006 and 2008.

^‡^%: Proportion of institutes that participated in survey of EQAS among institutes claiming medical care fee for HIV testing to HIRA.

* Number of institutes participating in the survey of External Quality Assurance Scheme (EQAS) for HIV testing.

University medical center: Hospital with greater than 100 beds that provide more than 8 services of medical examination of patients with an associated medical school.

General hospital: Hospital with greater than 100 beds that provide more than 6 services of medical examination of patients without an associated medical school.

Clinic center: Hospital with 30-100 beds.

Private clinic: Private clinic with less than 30 beds that provide medical examination primarily on an outpatient basis.

**Data from private clinical laboratory because HIV tests in private clinics were usually sent to private clinical laboratories.

Table 3 shows the estimated number of HIV-tested individuals and HIV seroprevalence in hospitals. A total of 2,662,715 individuals had HIV testing in hospitals in 2002. The estimated number increased to 4,774,625 individuals tested after ongoing annual increases. Women were tested more frequently (53%) than men (47%), and the aged 20-29 groups were higher in 2002 (21%) than in 2008 (16%, P = 0.0024), and the ≥60-year age group was elevated (P < 0.0001). Small towns showed an increasing proportion of tested individuals, as follows: 26% in 2002; 35% in 2005; and 39% in 2008 (P < 0.0001).

**Table 3:** Estimation of the number of HIV- tested individuals and HIV seroprevalence in hospital in Korea (2002-2008)

	2002		2003		2004		2005		2006		2007		2008	
**Category**	**No. of individuals (%)**	**SP (95%CI)**	**No. of individuals (%)**	**SP (95%CI)**	**No. of individuals (%)**	**SP (95%CI)**	**No. of individuals (%)**	**SP (95%CI)**	**No. of individuals (%)**	**SP (95%CI)**	**No. of individuals (%)**	**SP (95% CI)**	**No. of individuals (%)**	**SP (95% CI)**

Total	2662715	1.1 (1.0,1.3)	2942022	1.5 (1.3,1.6)	3152536	1.5 (1.3,1.6)	3655715	1.4 (1.3,1.6)	4288267	1.4 (1.2,1.5)	4559827	1.3 (1.2,1.4)	4774625	1.3 (1.2,1.4)

Gender														
Men	1241385(47)	2.2 (1.9,2.5)	1366003 (46)	3.0 (2.7,3.3)	1475469(47)	2.9 (2.6,3.1)	1725043(47)	2.9 (2.6,3.1)	2029604(47)	2.6 (2.4,2.8)	2157016(47)	2.5 (2.3,2.7)	2283012(48)	2.5 (2.3,2.7)
Women	1421330(53)	0.2 (0.1,0.3)	1576019(54)	0.2 (0.1,0.3)	1677067(53)	0.2 (0.2,0.3)	1930672(53)	0.1 (0.1,0.2)	2258663(53)	0.2 (0.2,0.3)	2402811(53)	0.2 (0.1,0.2)	2491613(52)	0.2 (0.1,0.2)

Age(years)														
<20	230182(14)	0.2 (0.0,0.3)	265790(14)	0.2 (0.0,0.4)	286719(14)	0.2 (0.0,0.3)	327129(14)	0.2 (0.1,0.4)	368356(14)	0.2 (0.1,0.4)	575860(13)	0.2 (0.1,0.3)	391628(13)	0.3 (0.1,0.4)
20-29	415085(21)	1.4 (1.0,1.7)	427927(20)	1.8 (1.4,2.2)	428347(18)	2.1 (1.7,2.6)	483521(17)	1.6 (1.2,2.0)	564369(17)	1.6 (1.3,2.0)	359699(17)	2.1 (1.7,2.6)	583999(16)	1.5 (1.2,1.8)
30-39	551075(20)	1.7 (1.3,2.0)	579531(19)	2.7 (2.3,3.1)	594234(18)	2.8 (2.4,3.2)	656387(18)	2.8 (2.4,3.2)	753751(17)	2.3 (2.0,2.7)	770464(17)	2.1 (1.8,2.5)	795353(16)	2.0 (1.7.,2.3)
40-49	527384(17)	1.6 (1.3,1.9)	579015(17)	2.0 (1.6,2.4)	609748(17)	1.9 (1.5,2.2)	698571(18)	1.8 (1.5,2.2)	816613(19)	1.8 (1.5,2.0)	754847(19)	2.0 (1.7,2.4)	867318(19)	2.2 (1.9,2.5)
50-59	379374(14)	1.2 (0.8,1.5)	429129(15)	1.3 (1.0,1.7)	465895(15)	1.2 (0.9,1.5)	560005(15)	1.6 (1.3,1.9)	682309(15)	1.4 (1.1,1.7)	855980(15)	1.1 (0.9,1.3)	802645(15)	1.5 (1.3,1.8)
60≤	559617(14)	0.3 (0.,0.5)	660629(15)	0.3 (0.2,0.5)	767593(17)	0.4 (0.3,0.5)	930102(18)	0.4 (0.3,0.5)	1102869(18)	0.6 (0.5,0.7)	1242976(20)	0.6 (0.5,0.7)	1333682(21)	0.4 (0.3,0.5)

Region														
Metropolitan	1 961671(74)	1.2 (1.0,1.3)	2 114283(72)	1.5 (1.3,1.6)	2155559(68)	1.6 (1.4,1.8)	2 368915(65)	1.4 (1.2,1.5)	2674029(62)	1.6 (1.4,1.7)	2799223(61)	1.6 (1.4,1.7)	2 917391(61)	1.6 (1.4,1.7)
Small town	701044(26)	1.0 (0.8,1.3)	827739(28)	1.4 (1.2,1.7)	996977(32)	1.2 (1.0,1.4)	1286800(35)	1.5 (1.3,1.7)	1614238(38)	1.0 (0.8,1.1)	1760604(39)	0.7 (0.6,0.8)	1 857234(39)	0.9 (0.8,1.1)

No. of individuals: Number of HIV-tested individuals, SP: HIV Seroprevalence per 10000 individuals, CI: Confidence Interval.

The annual HIV seroprevalence was between 1.5 and 1.3 per 10,000 individuals between 2003 and 2008; however, HIV seroprevalence was lower in 2002 than other years. Men had a higher seroprevalence than women and decreased from 3.0 per 10,000 individuals to 2.5 per 10,000 individuals (P = 0.0037); however, the seroprevalence in women (around 0.2 per 10,000 individuals) was stable between 2002 and 2008. The 30-39-year age group had the highest annual HIV seroprevalence, except in 2008, while the seroprevalence in the ≥60-year age group was increased, but there was no statistically significant difference (P = 0.0948). The annual HIV seroprevalence was higher in metropolitan areas than small towns, except in 2005.

## Discussion

The nationwide hospital HIV seroprevalence was calculated using a new estimation method in the present study. In 2008, the estimated number of HIV tests and tested individuals in hospitals increased and reached 5.0 million and 4.8 million cases, respectively. The annual HIV seroprevalence in hospitals was between 1.5 and 1.3 per 10,000 individuals during the study period, except 2002. The annual number of HIV-tested individuals in PHCs was approximately 0.3 million and the number in blood centers was approximately 1.6 million in 2008 [12]. These data led to an estimated 6.6 million as the total number of HIV-tested individuals, including the test-takers at hospitals in the same year. Thus, it is estimated that 13% of the Korean population (49 million) are tested annually [19]. Of HIV infections, 70% were diagnosed in hospitals, which performed 73% of HIV tests in Korea in 2008. The seroprevalence of HIV in hospitals was higher than the seroprevalence of HIV in blood centers and lower than PHCs when compared with previous results [13]. Most HIV infections diagnosed in hospitals were tested due to AIDS-related symptoms, pre-surgical evaluations and regular health check-ups [10]. The number of HIV-tested individuals increased from 0.35 million in 2002 to 1.5 million in 2008; the increased number was supported by annual data of tested individuals who sent insurance claims for HIV tests to HIRA. This result confirmed the increase in number of tested individuals per hospital and institution performing the tests. The main reason for increasing HIV-tested individuals may be the effect of advertisements for AIDS prevention through the mass media (initiated in 2004) and the official announcement of December as AIDS Test Days [20].

The highest insurance claim proportion for HIV tests in university medical centers is caused by the number of cases including operation, transplantation and severe diseases care which requires HIV test with insurance (Figure 1).

The seroprevalence in women was lower than men, while more women were tested than men. Women between 25 and 34 years of age were tested more frequently than any other age group. This was likely due to required HIV tests during pregnancy. Additionally, the proportion of women ≥ 60 years of age is higher than men, likely due to prolonged life spans in women [21].

We attempted to use statistical methods that would reduce errors since a perfect estimation without errors was not likely [4,22-24]. WHO/UNAIDS differentially applied the results from serologic surveys of specific groups, back-calculation methods, ratio methods, and multiplication of estimated annual AIDS cases by 20 according to factors, including year and population characteristics [4]. These estimation methods might be affected by data quality, representation rate, and modeling assumptions to result in errors. Therefore, estimation methods were improved by development of new methods (workbook method and special computer method) and tool standards to achieve superior estimations [22-24].

UNAIDS currently estimates the HIV prevalence of each country by reflecting population and characteristics through national surveillance and specific surveillance in high-risk groups [1,25,26]. This estimated that 13,000 HIV-infected individuals (95% CI, 7,500-42,000) have survived, and HIV prevalence in individuals 15-49 years of age was estimated at 0.040% (95% CI, 0.00-0.100) in Korea in 2007 [27]. However, we estimated that the nationwide HIV seroprevalence was 0.014% (95% CI, 0.011-0.016) after adjustment for age and gender in the same year. This seroprevalence was estimated for HIV seroprevalence of PHC visitors (0.043%; 95% CI, 0.027-0.060), blood donors (0.003%; 95% CI, 0.001-0.005) [13], and hospital visitors (0.015%; 95% CI, 0.012-0.018). The estimated HIV seroprevalence in this study was lower than UNAIDS estimation.

WHO/UNAIDS estimated current Korean HIV/AIDS data based on KCDC reports. This reflected some basic factors, including population, birth rate, death rate, and the number of HIV-infected individuals. However, it did not include advanced factors, such as current prevalence by institutional types or risk groups. The estimated value by the AIDS prediction model (EPP) will be most similar to the true value when it includes as many factors related to HIV prevalence as possible. Therefore, a survey by KCDC should be expanded to more factors affecting WHO/UNAIDS evaluation. UNAIDS estimation used only basic factors, while HIV seroprevalence in this study was estimated using information, such as data from EQAS survey, insurance claim data, and data for HIV tests in blood centers and PHCs. Because of this difference, we think that our results may be closer to the true value than UNAIDS estimation.

In our study, estimation of HIV-tested individuals using insurance data and hospital surveys can include several limitations. First, the number of surveys for clinic centers and private clinics are not as large as other hospital types. However, 12% of clinic centers are not too low by sampling methods for estimation [28]. Also, the number of tests in private clinics substituted from commercial clinical laboratories was likely accurate because most of the commercial clinical laboratories participated in the survey. Our results can include bias in insurance claim proportions for HIV tests by surveyed hospitals per year [29]. Second, there are some years in which surveys are missing. Without surveys, insurance claim proportions for HIV tests during 2002-2004 were estimated by that of 2005. Estimation of 2007 was reasonable because it was estimated as the mean of 2006 and 2008, but estimations for 2002 through 2004 appear to be biased and the value near year 2002 has more bias than the true value. There is a need to survey the number of HIV tests in hospitals per year to precisely estimate the insurance claim proportion for HIV tests. Finally, repeated values may be different between individuals with and without insurance claims. We are aware that there could be some bias in the prevalence estimated in the present study. Indeed, previous studies have identified potential biases in assessing the general population HIV prevalence from samples of selected groups such as prenatal clinic users, blood donors and hospital-based studies [30,31]. However, the repeated value for individuals with insurance claims was similar to the value of HIV-tested individuals during health check-ups in PHCs [11]. This report could enforce our assumption for the estimation method.

## Conclusions

The analytical adjustment using the estimation of insurance claim proportion and proportion of computerized insurance claims was applied to reduce errors in this study. The nationwide HIV seroprevalence and total number of HIV tests based in hospitals were also estimated using new methods developed and the seroprevalence trend was identified. This estimation may supply important basic data to revise AIDS strategies for better control.

## Competing interests

The authors declare that they have no competing interests.

## Authors' contributions

MKK and JHL designed and conceived the idea for the study and MKK supervised all aspects of its implementation and coordinated funding for the project. JHL completed the all data analyses and wrote the first draft of the manuscript. KJH contributed to the critical revising for important intellectual content and discussion. JSW contributed to collection EQAS survey data. SSK coordinated funding for the project and contributed to the critical revising for important intellectual content. All authors read and approved the final version of the manuscript as submitted to BMC Public Health.

## Pre-publication history

The pre-publication history for this paper can be accessed here:

http://www.biomedcentral.com/1471-2458/10/739/prepub
